# Transphyseal Separation of the Distal Humerus in a 5‐Year‐Old Child: A Rare Case Report

**DOI:** 10.1155/cro/9974926

**Published:** 2026-08-27

**Authors:** Sushil Pokhrel, Shivaji Karki, Sushil Sah, Ganga Tiwari

**Affiliations:** ^1^ Orthopedics Department, District Hospital Rukum, Musikot-Khalanga, Karnali, Nepal; ^2^ General Medicine, District Hospital Rukum, Musikot-Khalanga, Karnali, Nepal; ^3^ Health Administration, Health Service Directorate, Birendranagar, Karnali, Nepal

**Keywords:** case report, distal humerus, K-wire, pediatric elbow injury, transphyseal separation

## Abstract

**Background:**

Transphyseal separation of the distal humerus (TSDH) is an uncommon injury, typically seen in children under 3 years of age, with very few cases reported beyond this age group. Diagnosing this injury is challenging due to its cartilaginous nature. TSDH in infants is commonly treated with manual reduction and long‐arm cast application; however, a few case series have suggested that surgical treatment may be a better option in older children.

**Case Summary:**

A 5‐year‐old boy presented with left elbow swelling and restricted movements after a fall on an outstretched hand 2 days earlier. On physical examination, the three‐point bony relationship was maintained, and the distal neurovascular status was intact. Radiographs of the left elbow joint showed posteromedial displacement of the radioulnar complex with maintained radio‐capitellar line. The TSDH was suspected, and intraoperative arthrography was planned. The diagnosis of TSDH was confirmed intraoperatively with the aid of arthrography. The patient underwent closed reduction and percutaneous K‐wire fixation after confirming optimal alignment. Postoperatively, the elbow was immobilized using an above‐elbow posterior slab. At 5 weeks, the slab was removed, and gradual range‐of‐motion exercises were initiated. At the 2‐month follow‐up, the child had a satisfactory range of motion. At the 15‐month follow‐up, the patient demonstrated an excellent, pain‐free range of motion with no residual deformity or radiographic abnormalities.

**Conclusion:**

The TSDH in older children is a rare entity. The accurate reduction and timely diagnosis should be given priority so as to avoid late complications. If appropriate intervention is done ensuring adequate reduction and stable fixation, even with limited resources, excellent results can be achieved in such challenging cases.

## 1. Introduction

Transphyseal separation of the distal humerus (TSDH) is a rare pediatric elbow injury that can even be missed by experienced orthopedic surgeons. TSDH is often seen in children under 18 months of age and rarely in those older than 3 years [1]. DeLee et al. [2] described a type of TSDH that occurs between 3 and 7 years of age. TSDH can result from a fall or twisting injury at the zone of provisional calcification, which represents the transition between calcified and noncalcified portions of the growth plate [3]. Diagnosing this injury is challenging, even for experienced surgeons, due to its cartilaginous nature, and it is often mistaken for elbow dislocation [4].

Careful clinical examination helps differentiate a dislocated elbow from physeal separation of the distal humerus, as the three‐point bony relationship is preserved in physeal separation [4].

On plain radiography, the most common finding in TSDH is posteromedial displacement of the radioulnar complex relative to the humerus [5]. TSDH can be either a Salter–Harris Type I or Type II fracture [6]. Confirmation of TSDH can be done invasively with arthrography and non‐invasively with ultrasound or magnetic resonance imaging [7].

The commonly followed treatment modality in an infant is closed manual reduction with a long‐arm cast; however, studies have shown that surgical treatment often yields better outcomes in older children [8].

Although transphyseal separation is most common in children under 3 years of age, we report the successful treatment, with excellent outcome at 1 year follow‐up, of a rare case of modified DeLee Type C TSDH in a 5‐year‐old child with delayed presentation (> 48 h) following a fall injury on the left elbow. This article reports the case retrospectively.

## 2. Case Presentation

A 5‐year‐old boy sustained an injury to his left elbow after falling on his outstretched hand 2 days prior to presentation. He was referred to our center from a remote area in western Nepal for further evaluation.

The clinical examination revealed an edematous elbow joint without ecchymosis or external wound. The range of motion of the left elbow joint was restricted, but the three‐point bony relationship was maintained. The radial artery was palpable and distal median; ulnar and radial nerve functions were intact.

**Figure 1:**
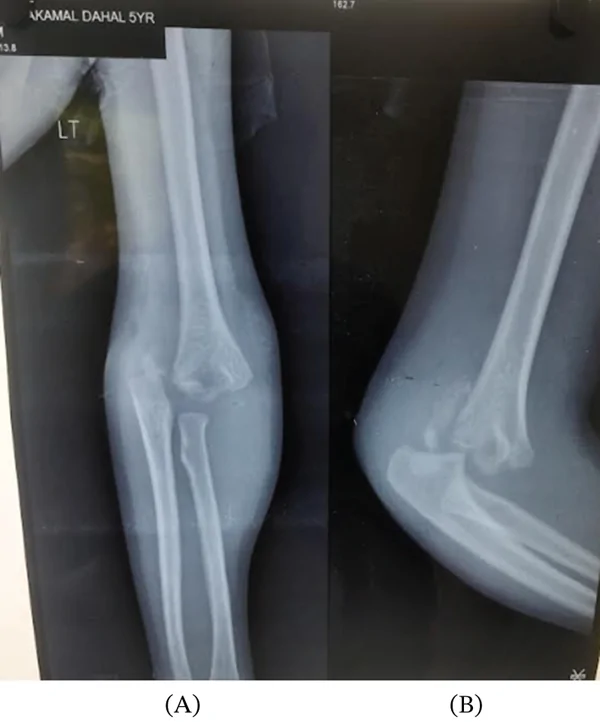
The preoperative x‐ray of the left elbow in extension showing antero‐posterior (AP) view and 90° lateral flexion (lateral view). The image (A) shows medial translation of the radioulnar complex in relation to the humerus with smooth metaphyseal border and image (B) shows postero‐lateral displacement of the capitellum along with the immature physis.

The radiological x‐ray imaging of the left elbow was done in antero‐posterior (AP) and lateral views. X‐ray imaging showed posteromedial displacement of the radioulnar complex while maintaining the anatomic relationship between the capitellar ossification center and the radial head/neck (i.e., the radio‐capitellar joint) with smooth metaphyseal border (Figure 1). The maintained radio‐capitellar joint differentiate the transphyseal separation from the elbow dislocation where the radio‐capitellar line is disrupted. The smooth metaphyseal border differentiated the transphyseal separation from the supracondylar fracture where the margins will be irregular. A fracture line extending from the lateral distal metaphysis through the physis to the articular surface with posterolateral displacement of fragment differentiated the lateral condyle fracture from the transphyseal separation, which was not present in this case. With this x‐ray findings, the presumptive diagnosis of transphyseal separation of distal humerus was made. In a rural setting such as ours, we did not have access to magnetic resonance imaging, the case was planned for fluoroscopy with arthrography and K‐wire fixation following closed or open reduction if required.

## 3. Surgical Procedure

Under brachial plexus block with intravenous anesthesia, the patient was placed in spine position. Sterile painting and draping were done, with the elbow placed on the arm rest in the abducted position. A 20‐gauge needle was inserted from the posterolateral aspect of the elbow directing medially and anteriorly towards the coronoid under fluoroscopy guidance. A 10 mL of dye was prepared by mixing 7 mL of Urografin 76% and 3 mL of sterile water. After confirmation with 1 mL of test dose of dye, an additional 3 mL of dye was injected into the joint and diagnosis of the TSDH was confirmed.

The manual closed reduction was performed with traction and countertraction with the elbow in 20° of flexion, followed by full elbow flexion in supination. Maintaining the elbow in a flexed position, again arthrography was done with 2 mL of dye for confirming the adequacy of reduction under fluoroscopy guidance.

**Figure 2:**
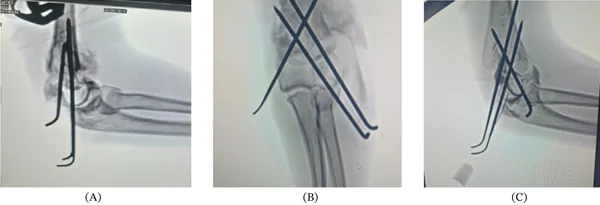
(A, B, C) The intraoperative fluoroscopic lateral (A), anteroposterior (B), and oblique (C) views of the elbow joint. The images illustrate the proper reduction of the physis in the sagittal and frontal planes. The reduction was maintained with 2 lateral and 1 medial K‐wires. The arthrography showed the joint was optimally reduced and the radiocapitellar line well maintained.

Two lateral K‐wires and one medial K‐wire were used for fixation (Figure 2). The medial K‐wire was placed to achieve greater medial stability. The medial K‐wire was inserted at approximately 45° of elbow flexion via a mini‐open approach to isolate and protect the ulnar nerve after placement of the lateral K‐wires. The stability of the fracture and positions of K‐wires were checked with dynamic fluoroscopy.

**Figure 3:**
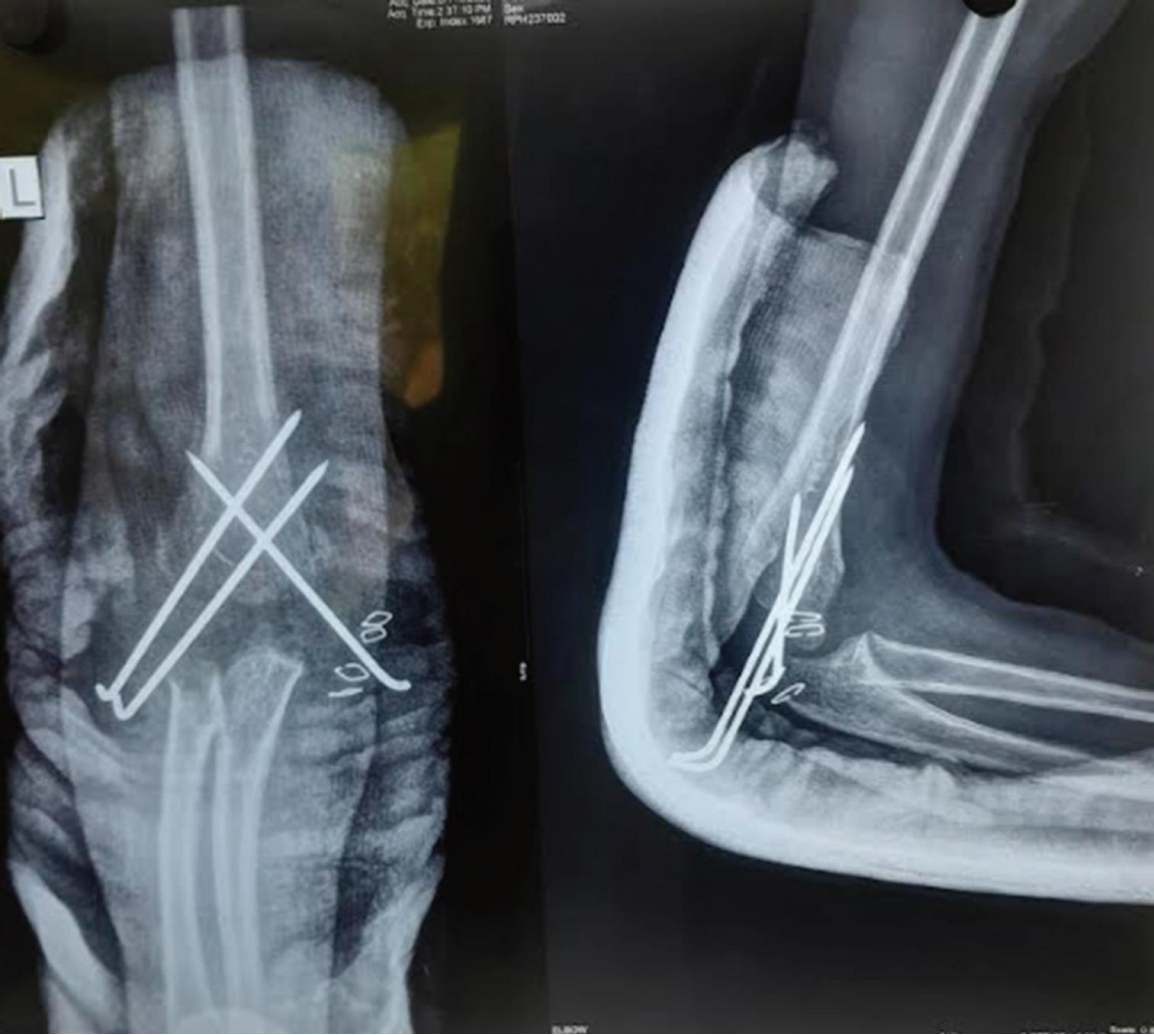
The second day postoperative x‐ray of the left elbow showing the AP and lateral views with the optimal reduction of the joint surface in frontal and sagittal plane after stabilizing the physis and capitellum with 3 K‐wires.

An above‐elbow posterior plaster‐of‐Paris slab was applied with the elbow in 90° flexion and the forearm in supination for immobilization. Postoperative x‐ray demonstrated satisfactory alignment (Figure 3).

The patient was discharged on the second postoperative day with oral cefuroxime for 10 days. Dressings were changed using 5% betadine and sterile gauze every 5 days to monitor for pin‐site infection until 5 weeks postoperatively. The staples at the medial pin site were removed on the 10th postoperative day. The posterior slab and K‐wires were removed under local anesthesia at 5 weeks. Range‐of‐motion exercises were gradually initiated, progressing from gentle passive to active movements over 2 months.

At the two‐month follow‐up, a goniometer was used for measuring the range of motion. The range of motion was 20°–110° of flexion, 0°–62° of pronation, and 0°–55° of supination, respectively. Although the range of motion was satisfactory, the child reported occasional pain over the lateral aspect of the elbow during extension.

**Figure 4:**
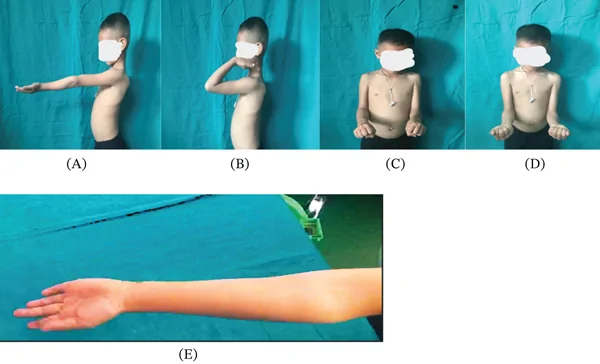
The images show the range of motion of the left elbow joint at 15‐month follow‐up: (A) extension from lateral views, (B) flexion, (C) pronation, (D) supination, and (E) extension from superior view, respectively.

**Figure 5:**
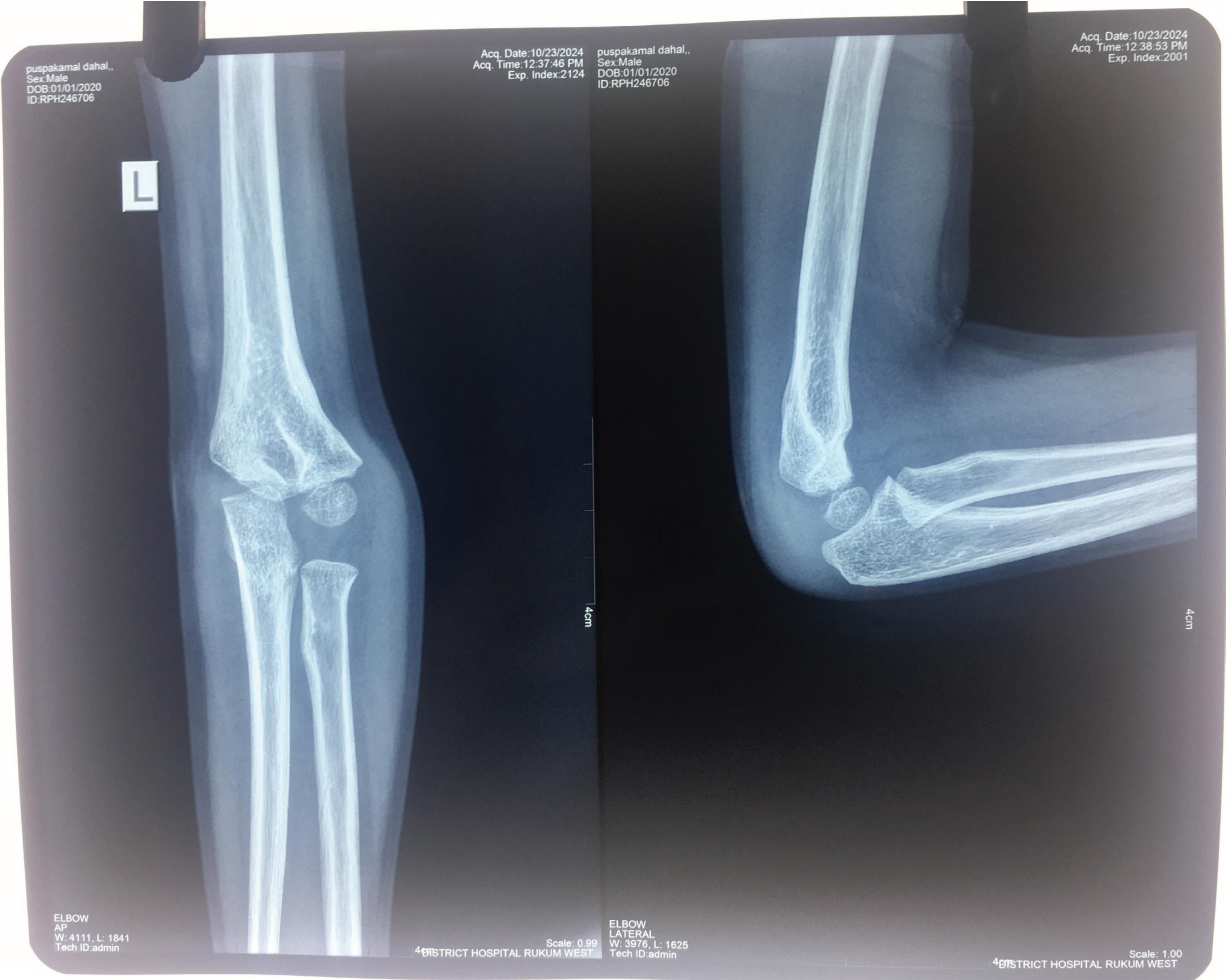
The image shows the x‐ray AP and lateral view of the left elbow at 15 months follow‐up. The ulno‐humeral angle (carrying angle ≈1°) is nearly straight, and there is no residual deformity.

The child was followed for 15 months to assess the range of motion and any visible deformity. The range of motion was assessed by using a goniometer. The final range of motion was 5°–145° of flexion with a 5° extension lag compared with the normal side (0°–145°). Supination and pronation were 0°–82° bilaterally. The carrying angle on the injured side was 1°, while it was 6° on the contralateral side. There were no complications such as cubitus varus deformity or persistent elbow pain. Radiologically, x‐ray AP and lateral views of the left elbow were done at 15 months to look for avascular necrosis of a medial condyle and cubitus varus deformity, which were absent in this case. The follow‐up range of motion and x‐rays at 15 months follow up are shown in Figures 4 and 5, respectively.

To categorize the outcome objectively, the child was assessed clinically for range of motion (functional factor) and radiologically for carrying angle (cosmetic factor) using Flynn′s criteria [9] (Table 1). According to Flynn′s criteria, the loss of motion and carrying angle was ≤ 5°, which was categorized as an excellent outcome in this child.

**Table 1:** Flynn′s criteria for assessing the range of motion and loss of carrying angle [9].

Result	Outcome	Rating cosmetic factor: carrying angle loss (degrees)	Functional factor: motion loss (degrees)
Satisfactory	Excellent	0–5	0–5
	Good	6–10	6–10
	Fair	11–15	11–15

Unsatisfactory	Poor	> 15	> 15

## 4. Discussion

Transphyseal injury is a rare condition, with case reports and series primarily describing its occurrence in newborns, infants, and toddlers up to 3 years of age. Some classifications, however, extend the age range to 7 years, although this is extremely uncommon after 3 years [2]. In our case, the patient was a 5‐year‐old child. Typically, TSDH occurs in newborns during forced obstetric maneuvers such as shoulder dystocia, although it has also been reported during cesarean section deliveries due to excessive traction [10]. In newborns and infants, longitudinal traction combined with twisting forces can cause transphyseal separation, as the physis represents the biomechanically weakest point of the distal humerus. Conversely, in older children, a fall onto an outstretched hand with the elbow extended may result in distal humeral fracture or physeal injury [10, 11].

Radiography is the initial imaging modality for elbow trauma. In cases of transphyseal separation, radiography may show misalignment between the ulna and humeral shaft on the anteroposterior view [12]. The most important radiographic finding is posteromedial displacement of the radius and ulna relative to the distal humerus [13]. Magnetic resonance imaging is the most accurate modality, providing multiplanar visualization of cartilage, bone, and soft tissues. However, the need for sedation and longer waiting times may delay treatment [14]. In our case, the presence of the capitellum facilitated the diagnosis.

DeLee et al. [2] initially classified this injury based on age, whereas Zhou et al. [15] later modified DeLee′s classification based on the presence of the secondary ossification center of the capitellum and the metaphyseal spike size. Based on these classifications, our case was categorized as Type C [2, 15].

Several studies have shown that closed reduction and above‐elbow cast application produce satisfactory outcomes in infants [16, 17]. However, evidence in older children is limited. Zhou et al. [15] recommended surgical management in older children, demonstrating that optimal reduction leads to better functional outcomes. Similarly, Tharakan et al. [18] suggested the use of intraoperative elbow arthrography to confirm adequate reduction and stabilization. Accordingly, we performed closed reduction and percutaneous pinning with intraoperative arthrography to confirm optimal reduction.

Several studies have reported that cubitus varus deformity is the most common complication during follow‐up, often resulting from avascular necrosis of the medial condyle [10, 19, 20]. In our case, no deformity was observed at the 15‐month follow‐up, and the outcome was excellent according to Flynn′s criteria. This finding is also supported by a recent systematic review by Chalidis et al. [21], which demonstrated that surgical intervention, either closed or open, reduces complication rates such as cubitus varus deformity.

## 5. Conclusion

The TSDH in older children is a rare entity. The accurate reduction and timely diagnosis should be given priority so as to avoid late complications. If appropriate intervention is done ensuring the adequate reduction and stable fixation, even with limited resources, excellent results can be achieved in such challenging cases.

## Funding

No funding was received for this manuscript.

## Conflicts of Interest

The authors declare no conflicts of interest.

## Data Availability

The data that support the findings of this study are available from the corresponding author upon reasonable request.
